# Supplementary material for: C‑Nucleosides Stabilize RNA by Reducing Nucleophilicity at 2′-OH
Source: ACS Cent Sci. 2025 Oct 28;11(12):2400–9. doi: 10.1021/acscentsci.5c01345 (PMC12746151; doi:10.1021/acscentsci.5c01345)
Supplement: Supplementary file 1 [file oc5c01345_si_001.pdf]

Supporting Information

C-Nucleosides Stabilize RNA by Reducing Nucleophilicity at 2'-OH

Dipanwita Banerjee,[1] Lu Xiao,[1] Pavitra S. Thacker,[1] Jayanta Kundu,[2] Muthiah Manoharan,[2] and Eric T. Kool[1*]

[1]Department of Chemistry, Stanford University, Stanford, California 94305, USA; [2]Alnylam Pharmaceuticals, Cambridge, MA 04142, USA

Contents

Materials and Methods………………………………………………………………… 2

Figures (S1-S12) ……………………………………………………………..… 8

Tables (S1-S3) ……………………………………………………………….…… 22

References ………………………………………………………………………..…… 26

Materials and Methods

Materials

eGFP mRNAs were purchased from TriLink Biotechnology. Short RNA oligonucleotides were purchased from IDT or synthesized by Stanford PAN facility unless otherwise stated (Supplementary Table S1). Purchased RNAs were diluted with nuclease-free water and concentrations measured with a Thermo Scientific Nanodrop spectrophotometer. All chemicals were purchased from commercial suppliers and used without further purification (Supplementary Table S2). All enzymes, kits, bio-reagents and software were obtained from the sources listed in Supplementary Table S2.

Accelerated mRNA ageing experiments

Two mRNAs (eGFP and m1ΨeGFP) were used to evaluate the effect of base modification on mRNA thermal stability. 20 μL of a solution containing 10 ng μL^{-1} of mRNA (30 μM) species in RNase-free water was incubated in a sealed RNase-free PCR tube at 37 °C. After the desired time, 3 μL of the mRNA solution was collected at each time point and RNA integrity was analysed by Capillary Electrophoresis (CE) on Agilent RNA 6000 Pico Chips using an Agilent 2100 Bioanalyzer. CE data were processed and visualized using 2100 Expert software (Agilent). CE traces are shown in Figure 2a. The apparent half-lives for spontaneous degradation were determined from the slope values of the linear relations by plotting the natural logarithm of the parent peak intensity from CE traces against time.

UV-melting experiments

UV absorbance was measured by using a Varian Cary 300 UV-visible spectrophotometer equipped with thermoprogrammer. The melting curves (absorbance versus temperature) of the oligonucleotides at 5 μM in 1x PBS buffer solutions without or with 10 mM $MgCl_2$ were obtained at 260 nm by annealing from 70 to 10 °C, followed by heating from 10 to 70 °C at a rate of 1 °C min^{-1}.

PAGE analysis of RNA spontaneous degradation

For kinetics assays of spontaneous degradation, RNA oligonucleotides (50-120 μM) were incubated for varied times in a reaction buffer containing a final concentration of 50 mM CHES buffer (pH 10.2) and 10 mM Mg^{2+}.[S1] We utilized a commercially available CHES buffer with a reported pH of 10.0 ± 0.15. Prior to initiating the experiments, the pH of the buffer solution was independently verified to be 10.19 at 37 °C. Temperatures were maintained at 37 °C unless otherwise indicated. Reaction aliquots (10 μL) at each time point were quenched by diluting into 2.5 μL 1 M Tris-HCl (pH 7.0) and 1 μL 0.5 M EDTA. To ensure accurate quantification of the remaining RNA fraction at each time point, reaction mixtures were gently vortexed and centrifuged prior to quenching. Quenched samples were further diluted (2-fold) by adding to 8 M urea loading buffer with dye and stored at –80 °C prior to gel loading. Reaction mixtures were separated by 20% denaturing PAGE in 1x TBE buffer (pH 8.3) at 25 mA for 1 h and the gel image was acquired by iBright 1500 imager after staining with SYBR-gold. After converting images to 16-bit using ImageJ software, analysis was done by plotting band intensities from same sized rectangular area on selected bands. Quantified band intensities were used to calculate remaining RNA fractions for measuring kinetic parameters.

RNA enzymatic degradations

For kinetics assays of enzymatic degradation, RNA oligonucleotides (100 μM) were incubated at 37 °C for 10 min in 30 μL reaction buffer containing 100 mM Tris-HCl buffer (pH 7.4). 30 μL of 20 ng mL^{-1} RNase A solution (0.73 nM) was then added and the mixture incubated for varied reaction time. Reaction aliquot (10 μL) at each time point was quenched by diluting into 1 μL RNaseOUT^{TM} (40 U μL^{-1}), 1 μL of 0.5 M EDTA, and 1 μL of 100 mM DTT. Same method was followed for other enzymatic degradations, where 30 μL of 0.02 U μL^{-1} or 2.0 U μL^{-1} ribonuclease RNase 1 and RNase 4, respectively, was used to start the reaction. Quenched samples were further diluted (2-fold) by adding to 8 M urea loading buffer with dye and storing at –80 °C prior to gel loading. Reaction mixtures were separated by 20% denaturing PAGE and analyzed by using ImageJ software following the method described above. The rate constants of pseudo-first-order ($k_{clv(app)}$) and second-order (k_2) kinetics were quantified (Table S3) following the method described below.

Determination of rate constants

Rate constants were determined under various reaction conditions by first plotting the natural logarithm of the fraction of substrate remaining uncleaved at different incubation times. The negative slope of the resulting line was generated by a least-squares fit to the data, reflecting the rate constant for RNA cleavage by transesterification. Data reported are averages of at least three separate experiments.

CD measurements

Circular Dichroism (CD) spectra were obtained using a JASCO-820 spectropolarimeter with a temperature controller. The CD spectra were collected from 350 to 200 nm wavelength at a scan rate of 50 nm min^{-1} in 0.1 cm path-length cuvette at 4 °C. All spectra were measured at a strand concentration of 20 μM in 1x PBS buffer (pH 7.4) solution with 10 mM $MgCl_2$. The cuvette-holding chamber was continuously flushed with a stream of dry N_2 gas to avoid water condensation on the cuvette exterior.

Synthesis of acylimidazole reagents

Reagents (NAI and meNAI) were synthesized following the general protocol.[S2,3] The carboxylic acid precursor (1.0 equiv.) was dissolved in dry DMSO as a 4 M solution. To it was added an equal volume of suspension containing 1.0 equiv. 1,1′-carbonyldiimidazole in dry DMSO at room temperature. The resulting solution was stirred at room temperature for 1 (NAI) or 3-4 h (meNAI). The completion of reaction was monitored by ^{1}H NMR spectroscopy, which confirmed a 1:1 mixture of the acylimidazole and free imidazole. After the reaction, the crude solutions were used as a 2 M acylimidazole stock solution without further purification.

Kinetics studies of RNA acylation

1.0 μL of RNA stock (100 μM) was dissolved in 8.5 μL water solution with 1x PBS buffer (pH 7.4) and incubated at 37 °C for 15 min. Then, 0.5 μL freshly prepared 2 M acylating reagent was added to RNA solution to kept DMSO 5%. The reaction was incubated at 37 °C for the

indicated time and quenched by diluting with RNase-free water to 500 μL. The mixture of RNA and acylated RNA was purified with Amicon ultra centrifugal filtration (3K) and stored at –80 °C. The ratio of substrate and product in the RNA mixture (as peak intensities) was analyzed with MALDI-TOF operating in the linear negative mode in 3-HPA matrix.

Mass spectrometry

The MALDI-TOF mass spectra were recorded on a Bruker Daltonik Microflex MALDI-TOF spectrometer with a N_2 laser, in linear negative mode. An MSP BigAnchor 96 ground steel target was used for MALDI-TOF analysis. For the linear negative mode, a mixture of 0.3 M trihydroxyacetophenone in EtOH and 0.1 M aqueous ammonium citrate (2:1) used as matrix/comatrix, and always freshly prepared before analysis. Spectral data were analyzed with Flex Analysis, version 3.4 (Bruker).

The pK_a determination of alcohols

Four alcohols (2,2,2-trifluoroethanol, 2,2,2-trichloroethanol, 2,2-dichloroethanol, 2-chloroethanol were selected to study acylation reaction. The pK_a values of selected alcohols were reported as 12.4, 12.2, 13.1, and 14.3, respectively.[S4] Reported pK_a values were used in this study.

Kinetics study for the acylation of alcohols

1 M alcohol solutions were prepared with selected alcohols in D_2O. For 200 μL reaction mixture, 20-40 μL 1 M alcohol solution was mixed with D_2O and 10 μL solution of 20x PBS buffer (pD 7.4) prepared in D_2O. The mixture was incubated at 37 °C or 25 °C for 15 min. Then, to the mixture 10-20 μL 2 M stock solution of the acylating reagents in DMSO-D_6 was added to react with alcohols in 1:1 ratio and kept DMSO 5-10%. The reaction was incubated at 37 °C or 25 °C for the indicated time and quenched by diluting with RNase-free water to 700 μL. The ^{1}H-NMR of reaction mixture was immediately recorded after quenching the reaction and analyzed the reaction rate from the ratio of substrate and product in the reaction mixture (as peak intensities).

NMR spectroscopy

^{1}H NMR spectra were recorded on Varian Mercury 500 MHz NMR spectrometer. Spectra were internally referenced to the residual solvent signal.

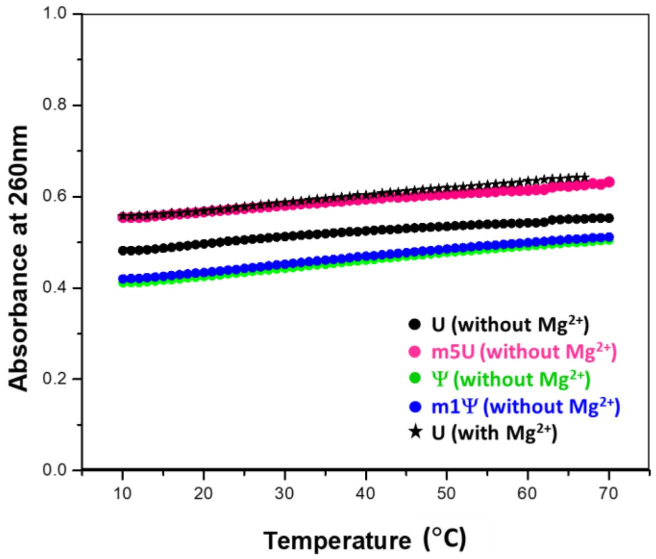

Figure S1. UV thermal denaturation plots at 5 μM concentration for A oligonucleotides containing U (black), m5U (pink), Ψ (green), and m1Ψ (blue) in 1x PBS buffer solutions without (circle) and with (star) 10 mM $MgCl_2$.

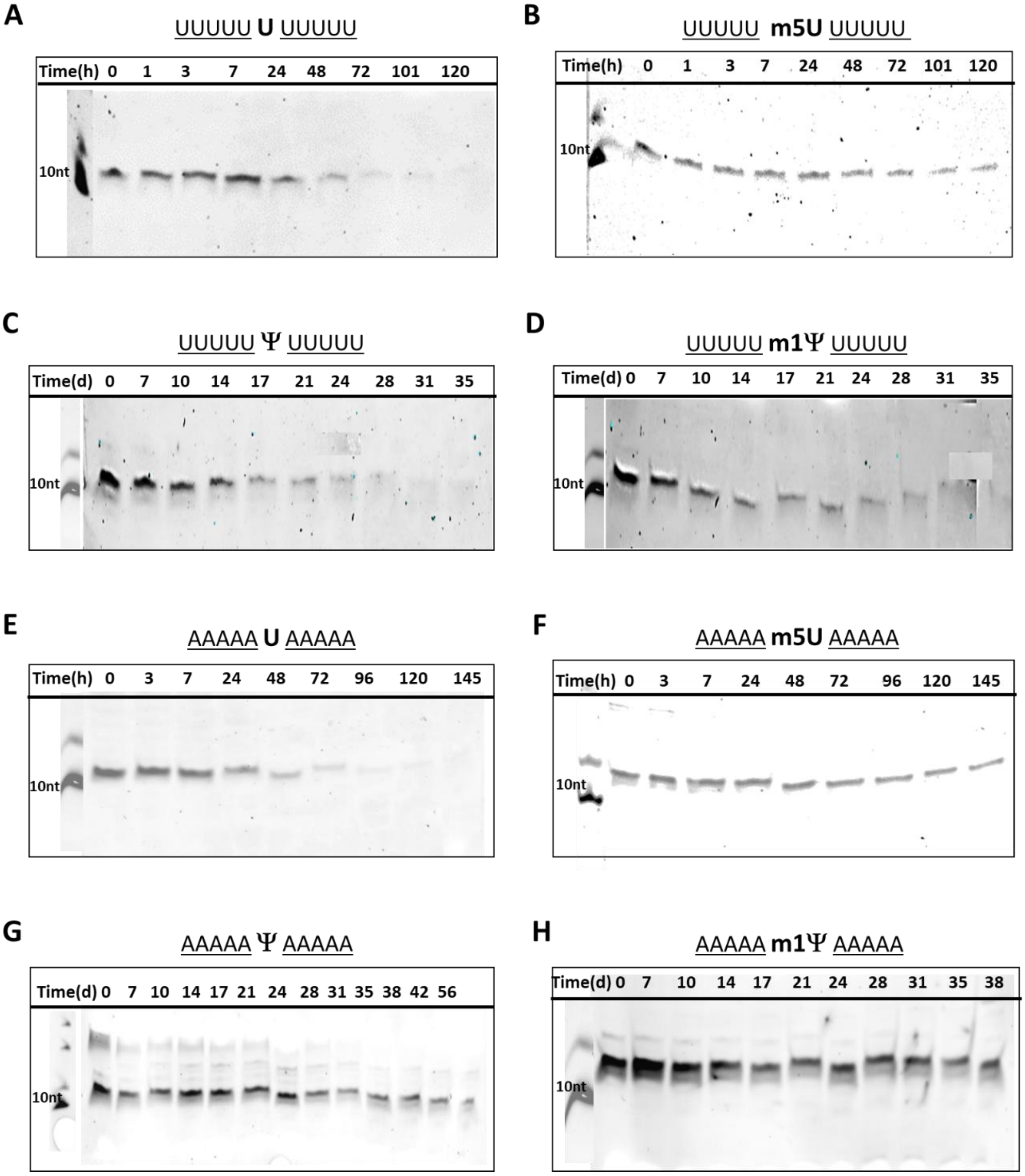

Figure S2. Representative gel images (20% denaturing PAGE) for spontaneous degradation of U, m5U, Ψ, and m1Ψ in U contexts (A, B, C, D respectively) U and A contexts (E-H, respectively) after incubating substrate oligonucleotides in CHES buffer (pH 10.2) solution with Mg^{2+} at 37 °C for the noted time period.

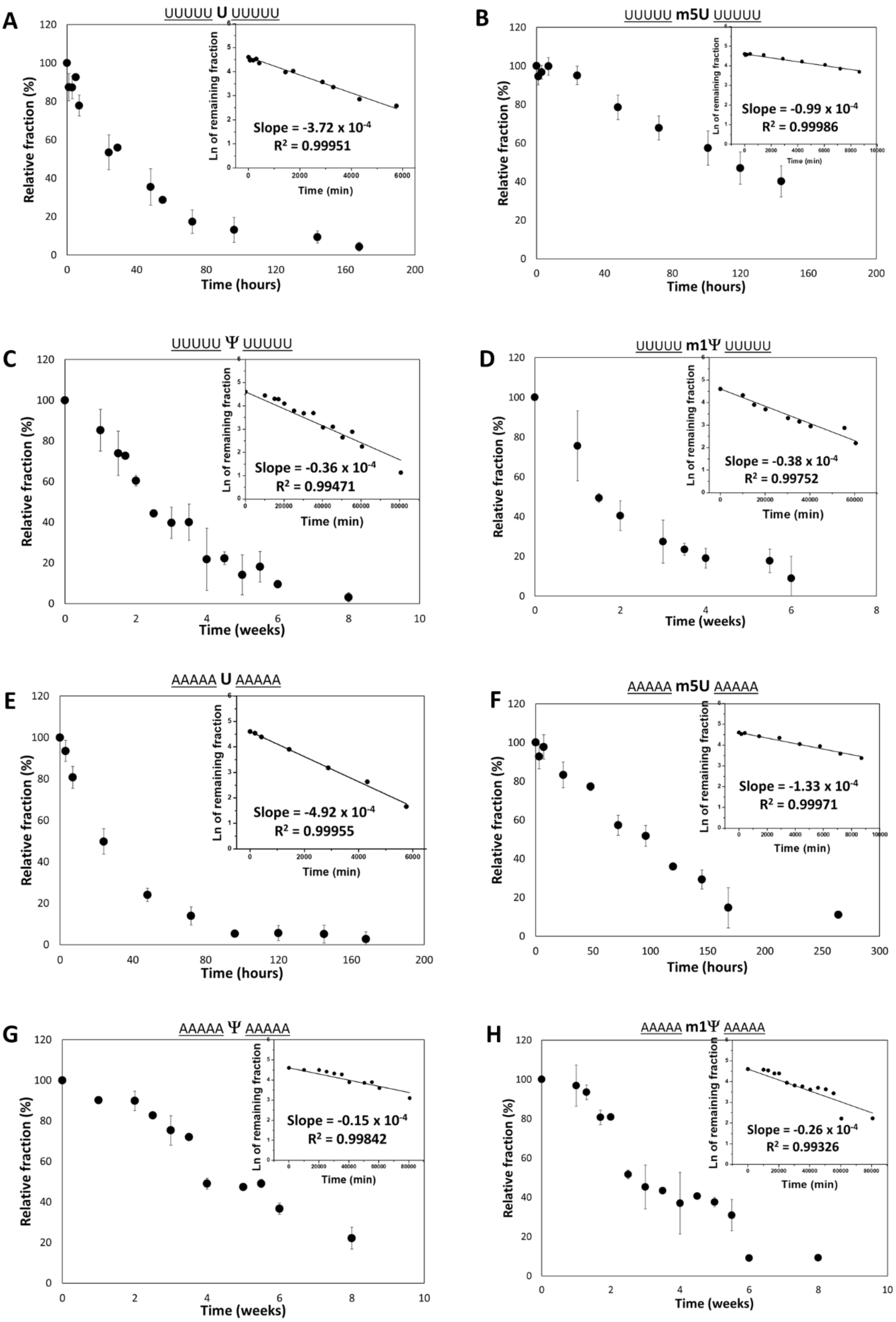

Figure S3. Plots of the decay of the central RNA linkage for U, m5U, Ψ, and m1Ψ in U contexts (A, B, C, and D, respectively) and A contexts (E, F, G, and H, respectively). The relative intensity of bands was derived from gel analysis (see examples in Fig. S2). Negative slope of each linear plot reflects the rate constant k_{clv} (min^{-1}) for spontaneous degradation of the oligonucleotide in the CHES buffer (pH 10.2) at 37 °C.

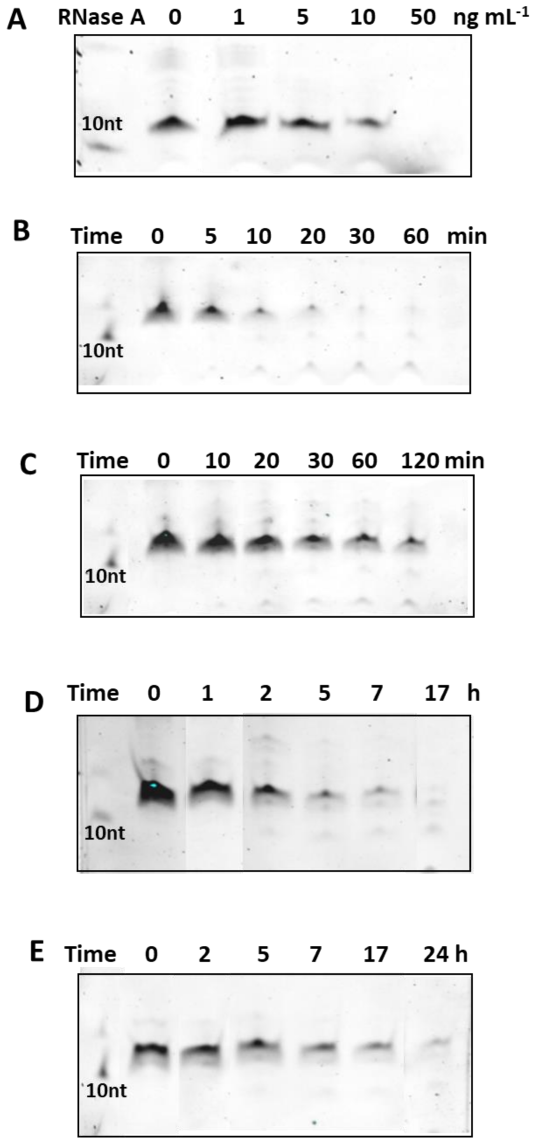

Figure S4. A. Representative denaturing 20% PAGE image analyzing ribonuclease A catalyzed cleavage for screening of RNase A concentrations as shown, with 50 µM unmodified A context oligonucleotide using RNase A at 0 to 50 ng mL^{-1} concentration in final reaction mixture after 10 min reaction. B, C, D, E. Representative images of 20% denaturing PAGE gels for for enzymatic degradation of U, m5U, Ψ, and m1Ψ, respectively, in A context after incubating substrate oligonucleotides with 10 ng mL^{-1} in Tris-HCl buffer (pH 7.4) solution at 37 °C for the noted time.

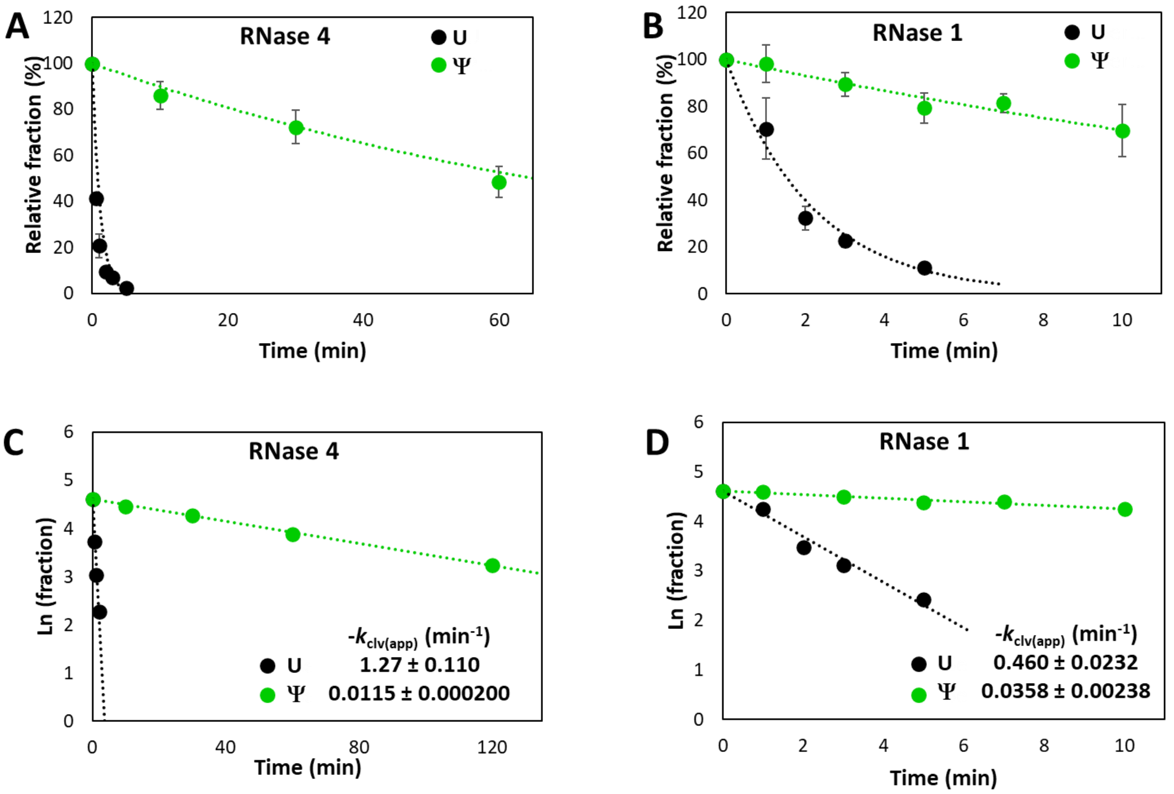

Figure S5. (A),(B) Plots showing decay of the central phosphodiester bond of RNAs (50 μM) by enzyme-catalyzed cleavage at 37 °C with RNase 4 (1.0 U μL^{-1}) and RNase 1 (0.01 U μL^{-1}), respectively, for 11 nt A-context oligonucleotides containing a central U and Ψ with time. (C),(D) Linear plots of Ln(remaining RNA fraction) vs time for unmodified and modified uridine-containing RNAs. Pseudo-first-order rate constants ($k_{clv(app)}$) are derived from the negative slopes. Data were obtained from 3 replicates with error bars showing standard deviations.

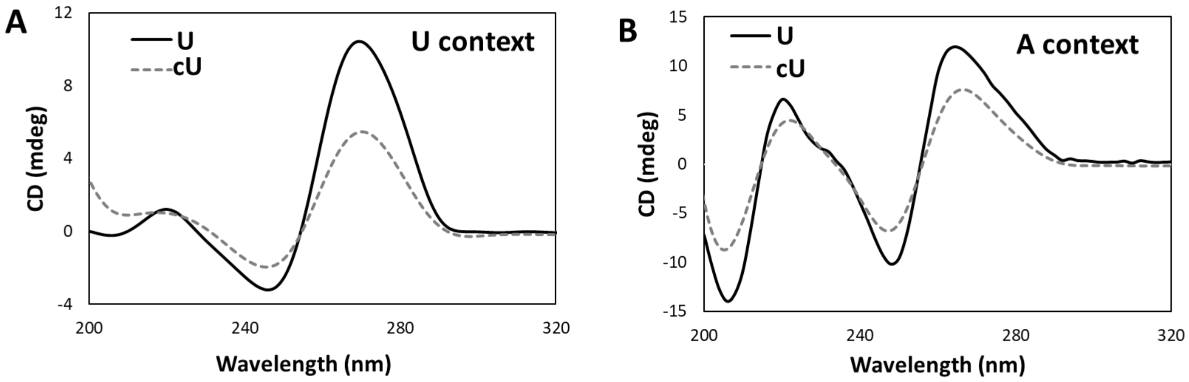

Figure S6. Comparison of CD spectra of U and A oligonucleotides, respectively, containing cU modification with corresponding unmodified oligonucleotides of 20 µM concentration at 4 °C in the 1xPBS buffer (pH 7.4) containing 10 mM Mg^{2+}.

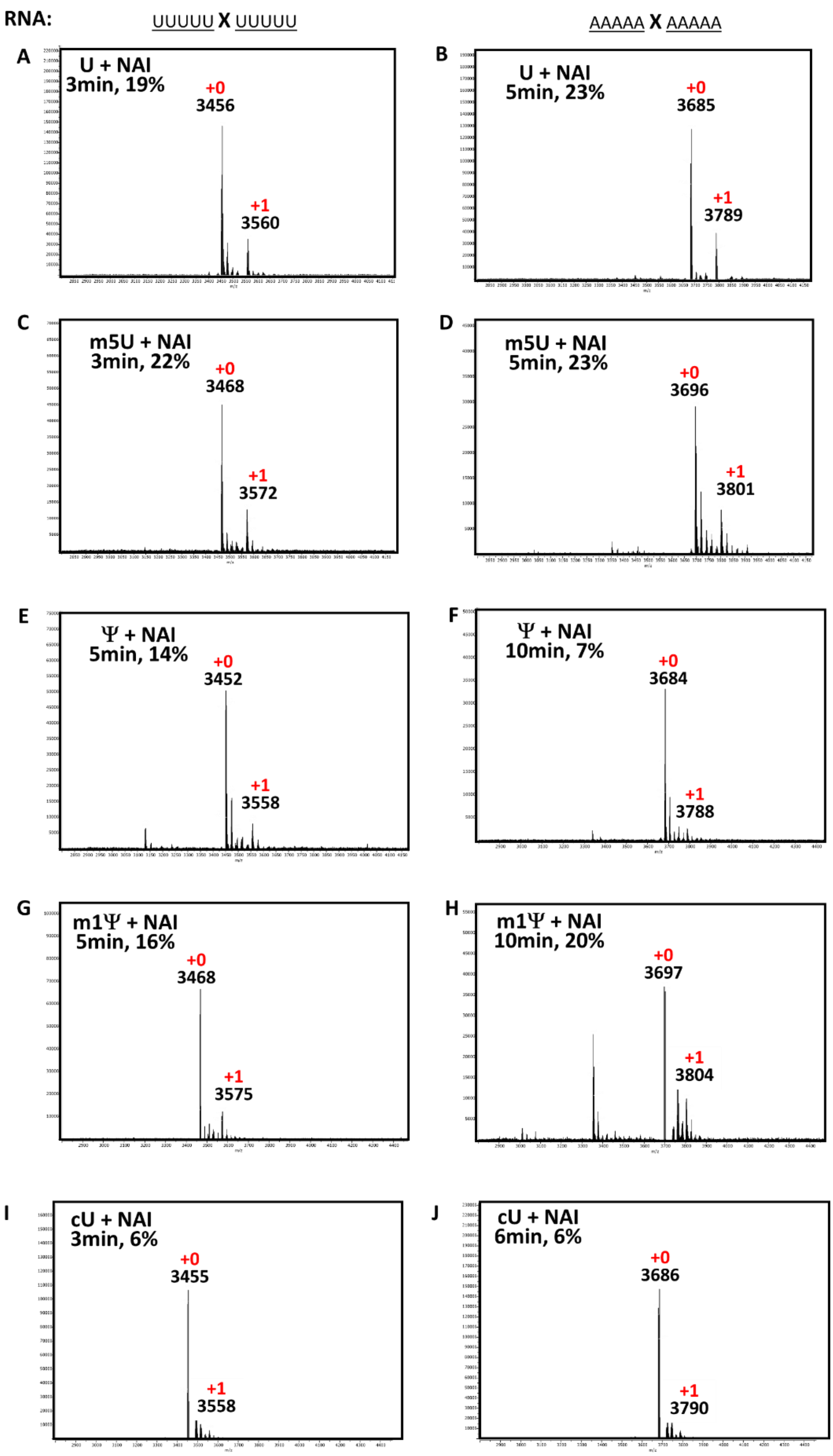

Figure S7. Representative MALDI-TOF spectra after indicated times of acylation reactions with NAI (100 mM) for RNA oligomers (10 μM) with uridine modifications (X), such as U, m5U, Ψ, m1Ψ, and cU in (A, C, E, G, and I, respectively) U and (B, D, F. H, and J, respectively) A contexts at 37 °C to evaluate kinetics of 2'-OH reactivity shown in Table 2.

^{1}H NMR spectrum of TFE after 2min reaction (25% conversion)

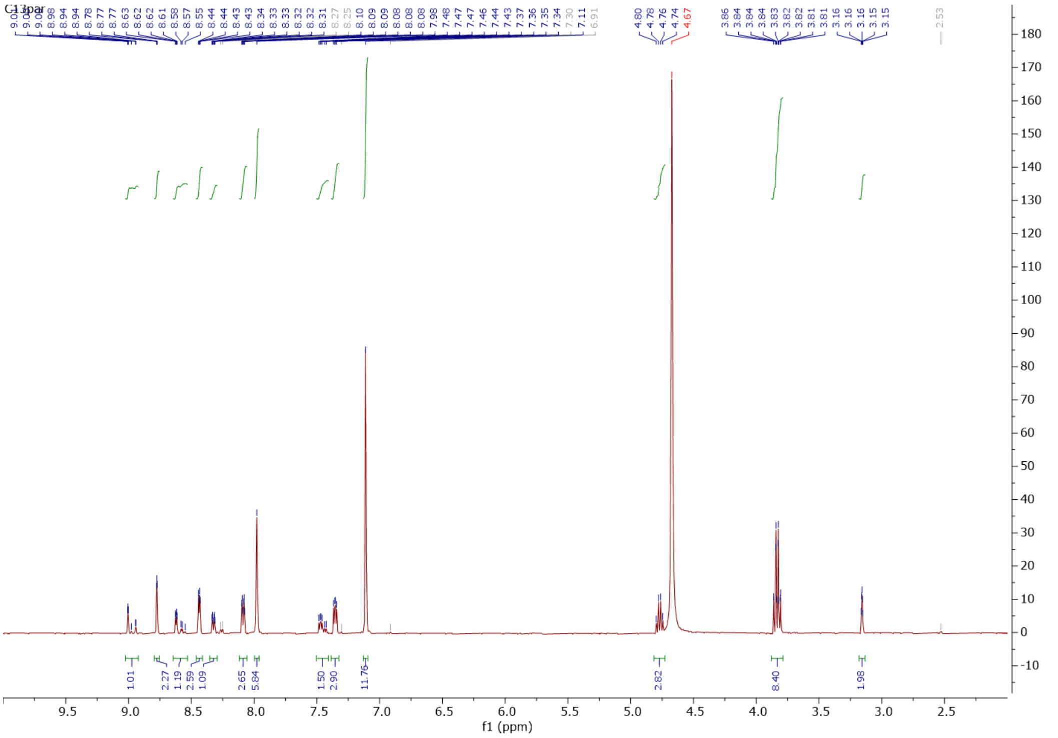

^{1}H NMR spectrum of TCE after 3min reaction (13% conversion)

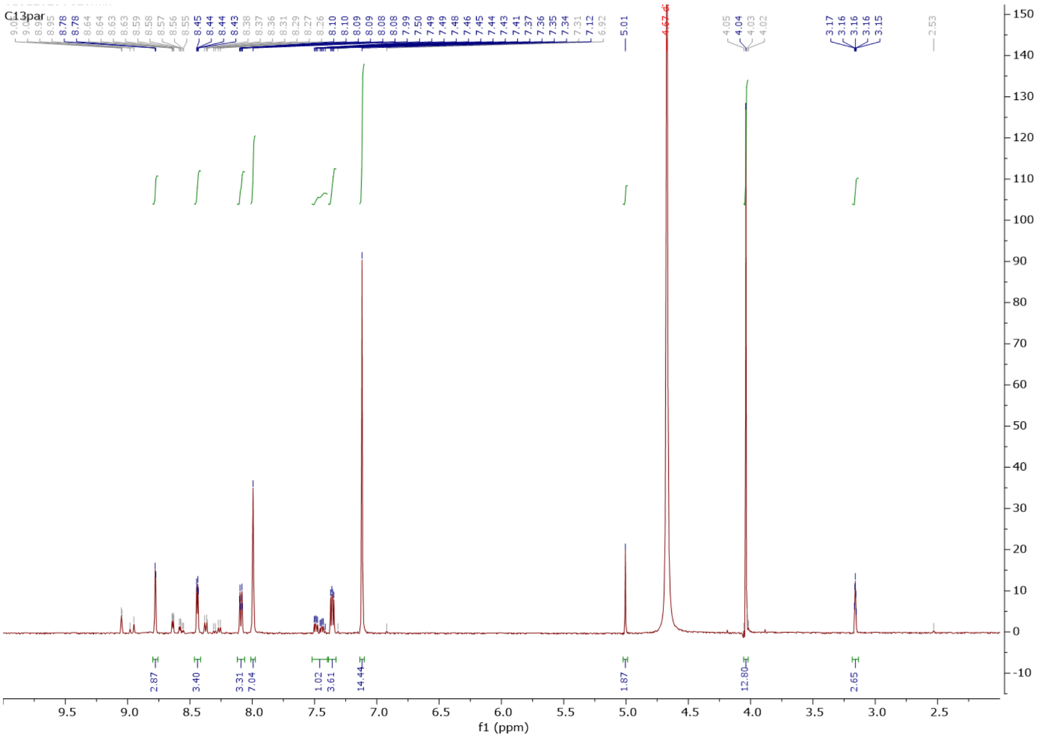

(Figure S8 continued)

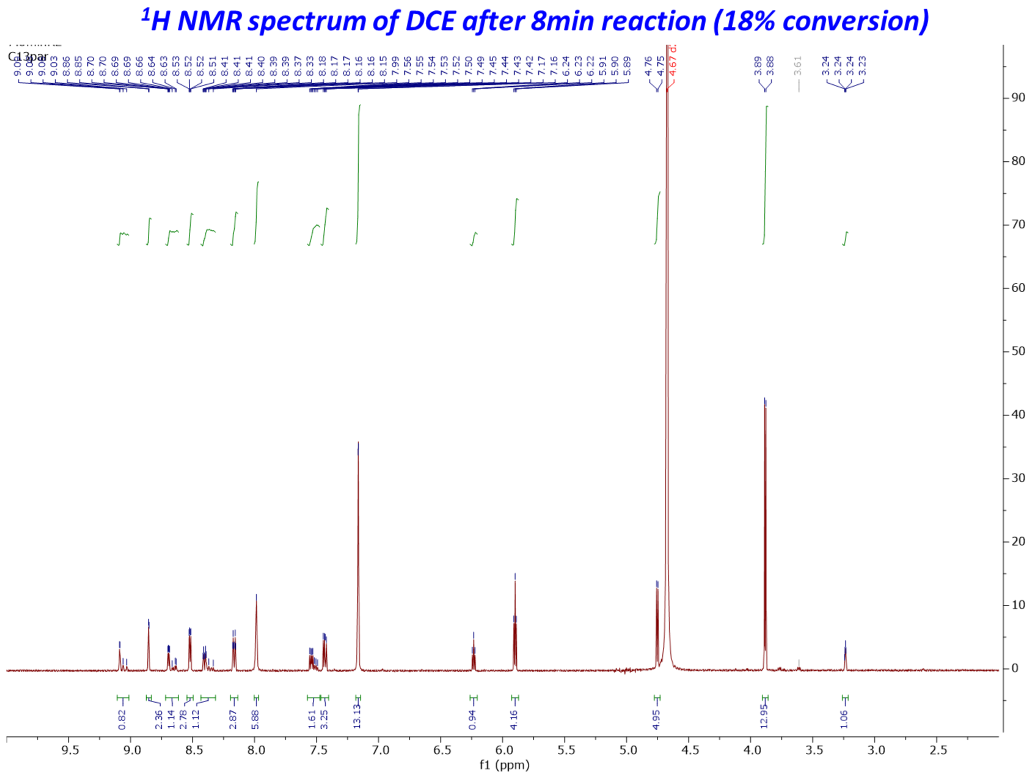

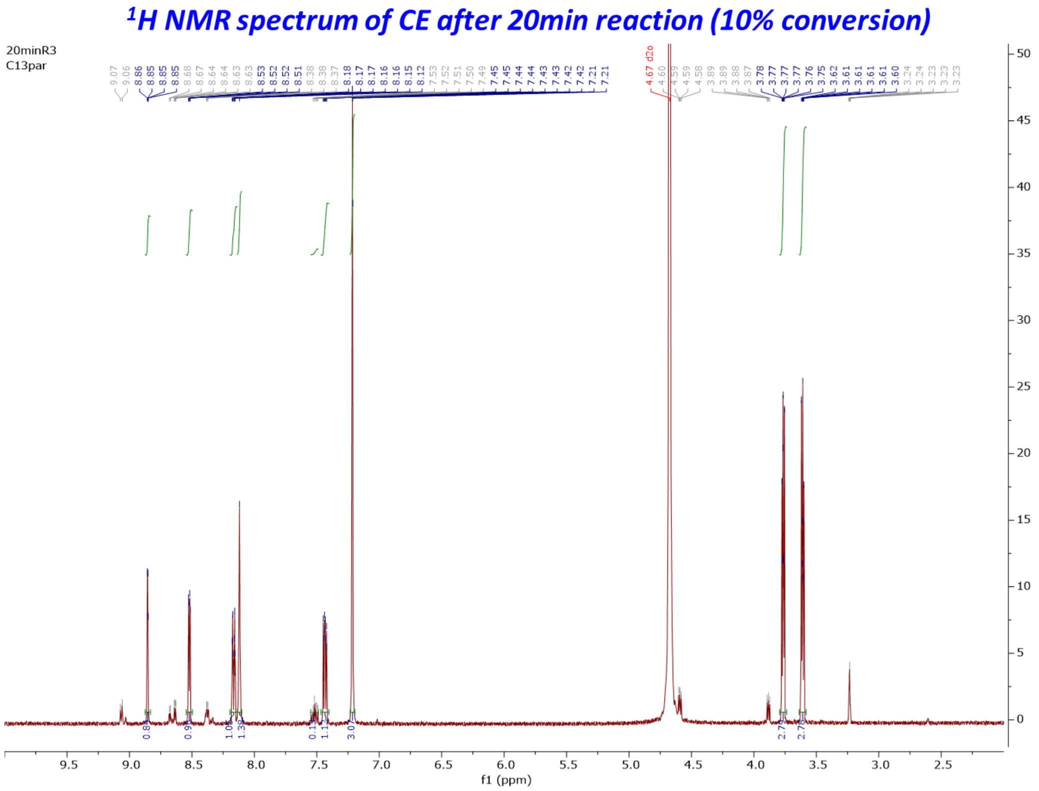

Figure S8. Representative ^{1}H NMR spectra after indicated time of acylation reaction with NAI and alcohols (1:1) at 37 °C to evaluate kinetics of 2′-OH acylation. 2,2,2-trifluoroethanol, 2,2,2-trichloroethanol, 2,2-dichloroethanol, and 2-chloroethanol alcohols are written as TFE, TCE, DCE, and CE, respectively. The intensities of corresponding peaks of substrate and products were used to analyze rate constants of second-order kinetics in Table 3 and Figure S8.

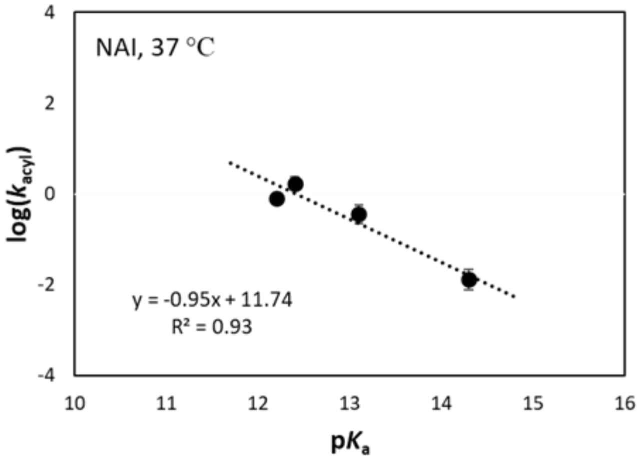

Figure S9. Plot of log values for the acylation rate constants (k_{acyl}) with NAI versus pK_a for primary alcohols (see Figure 5E) (black circles) at 37 °C for evaluating the contribution of pK_a shift to the reactivity of hydroxyl groups.

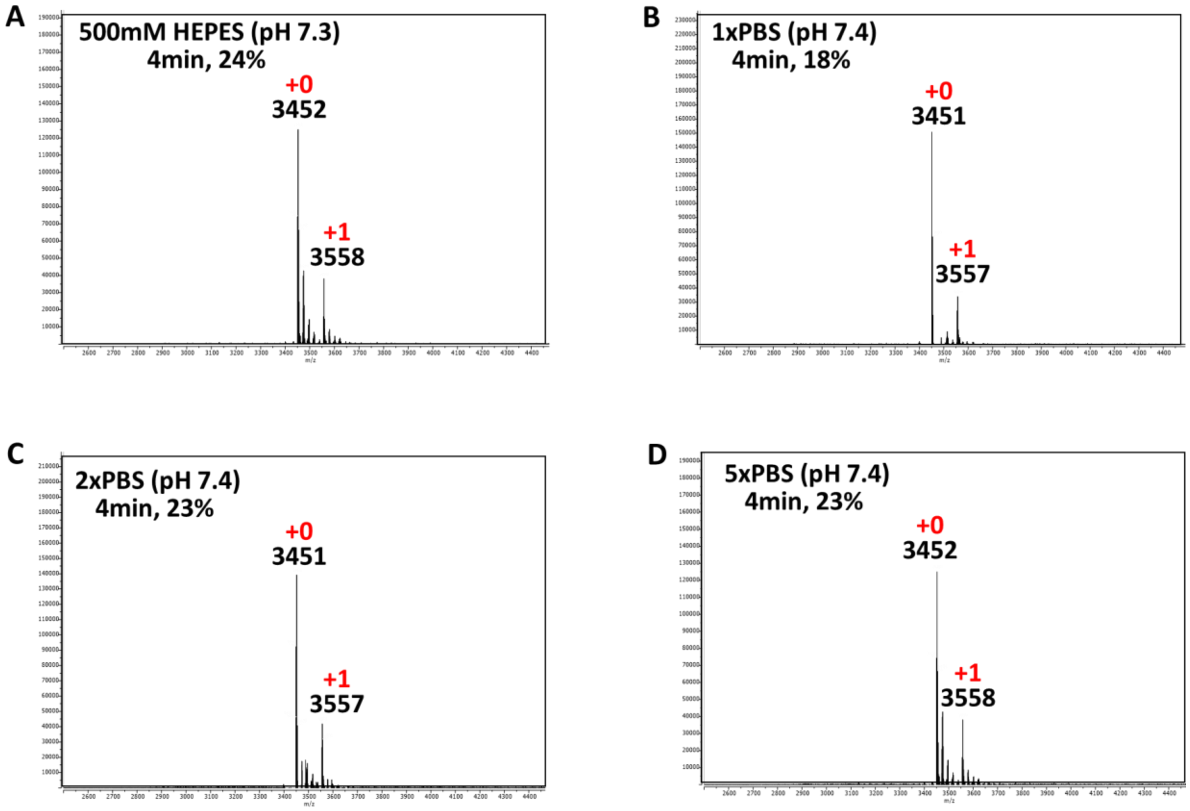

Figure S10. Representative MALDI-TOF spectra of after 4 min of acylation reaction with NAI (100 mM) for unmodified U context (10 μM) at 37 °C in the solution of A. 500 mM HEPES (pH 7.3), B. 1x, C. 2x, and D. 5x PBS buffer (pH 7.4), showing independency of 2'-OH acylation reaction on the buffers.

^{1}H NMR spectrum of TFE after 4min reaction (40% conversion)

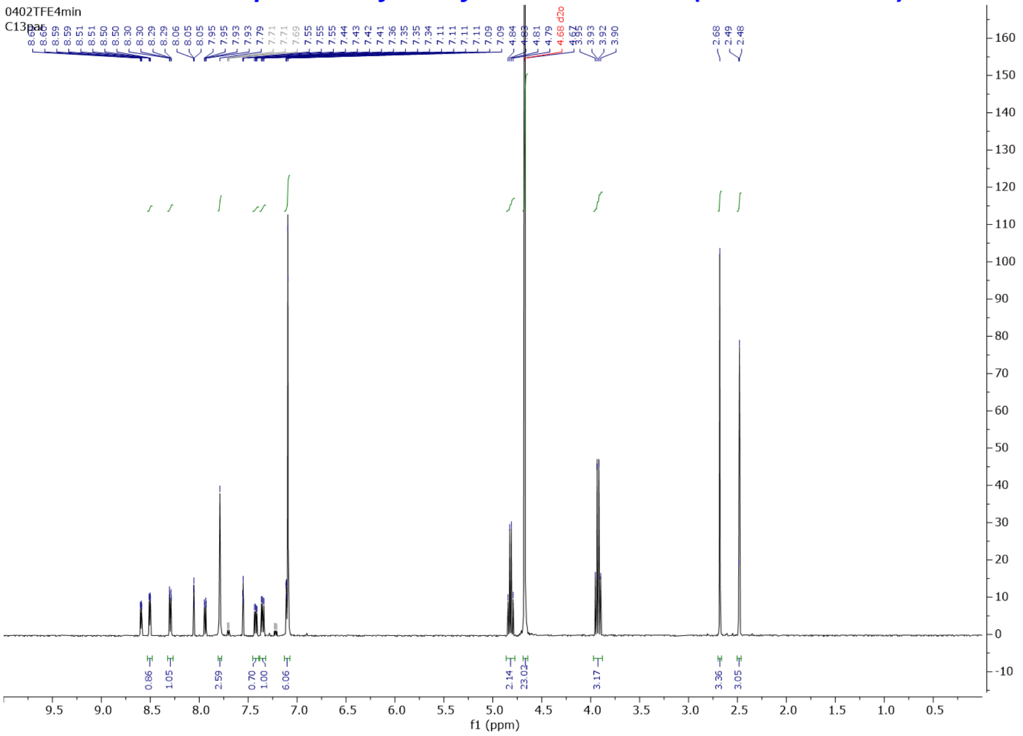

^{1}H NMR spectrum of TCE after 6min reaction (31% conversion)

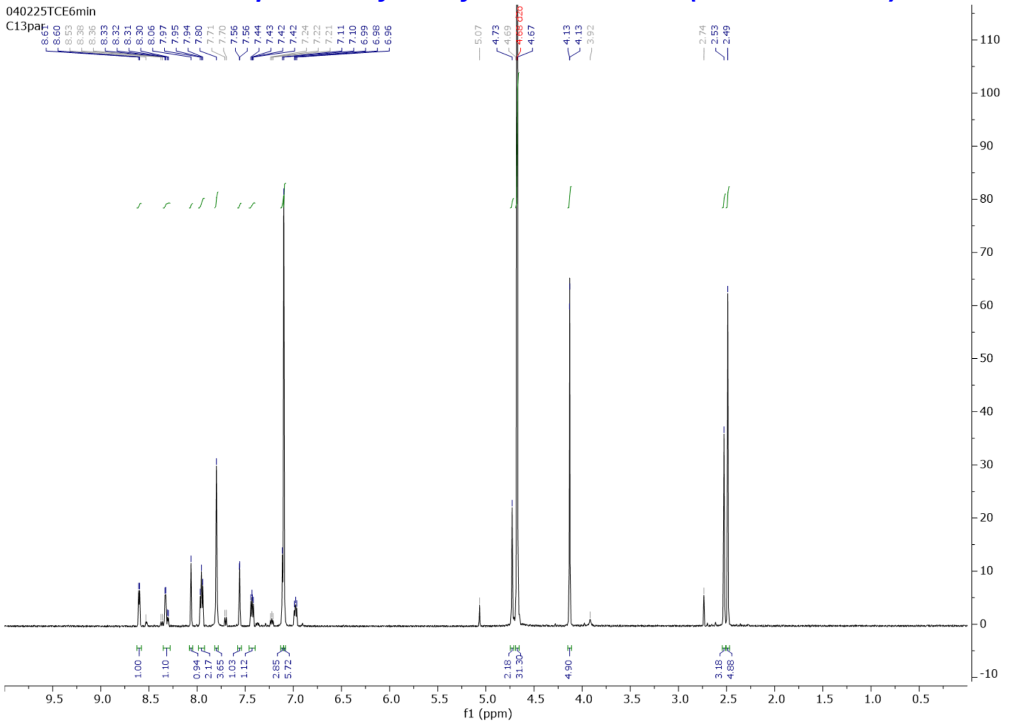

(Figure S11 continued)

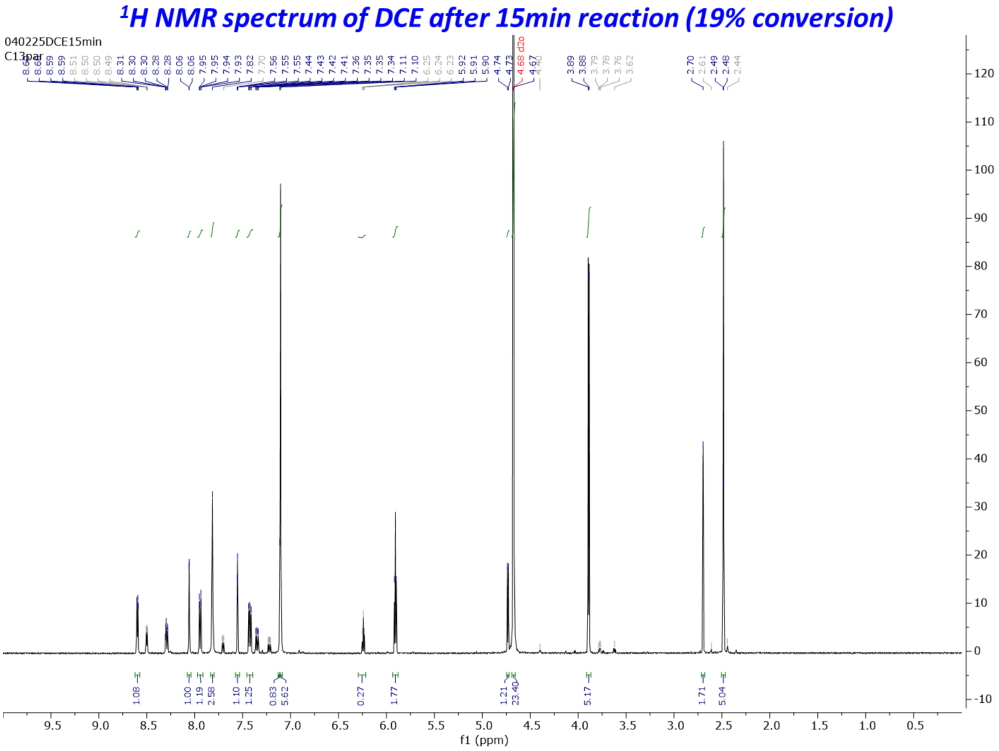

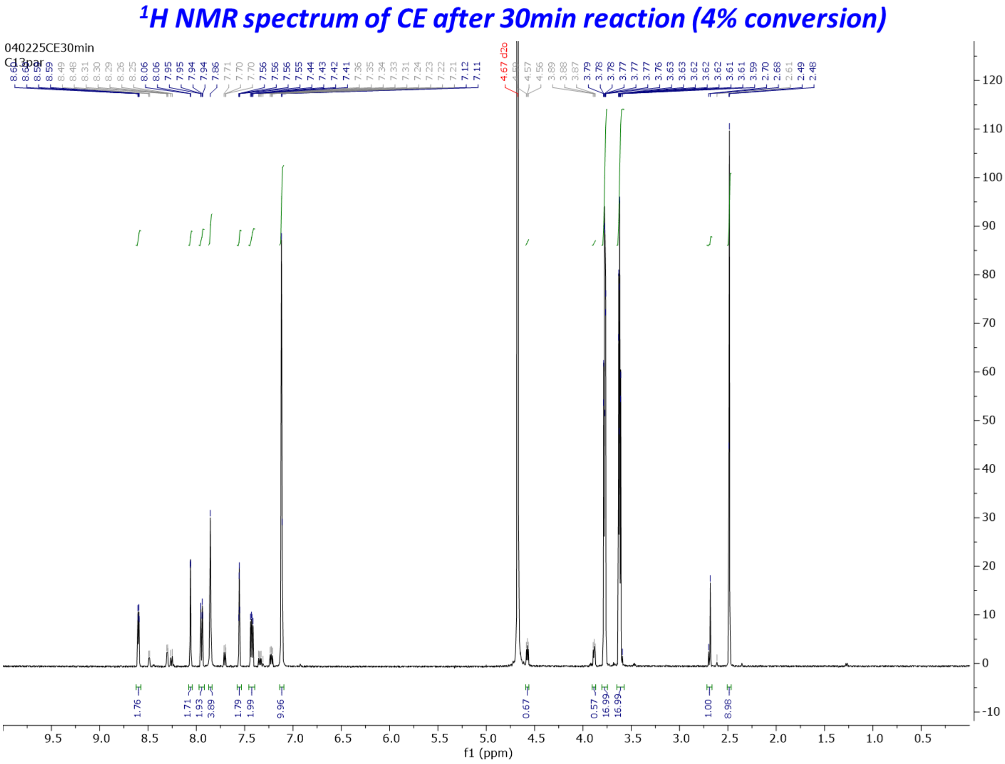

Figure S11. Representative ^{1}H NMR spectra after indicated times of acylation reaction with meNAI and alcohols (1:1) at 25 °C to evaluate kinetic order of corresponding OH acylation. Here, 2,2,2-trifluoroethanol, 2,2,2-trichloroethanol, 2,2-dichloroethanol, and 2-chloroethanol alcohols are written as TFE, TCE, DCE, and CE, respectively. The intensities of corresponding peaks of substrate and products were used to analyze rate constant of second-order kinetics in Table 3 and Figure 6C.

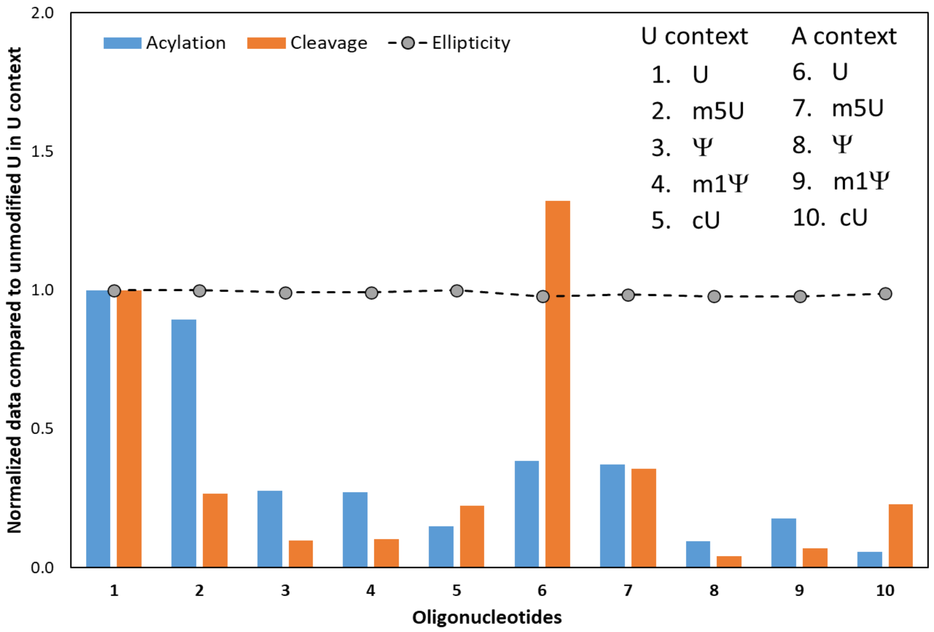

Figure S12. Comparison of normalized rate constants for the acylation and spontaneous cleavage of modified nucleotides in the RNA oligonucleotides as shown in Figure 5B with corresponding ellipticity shift. Normalization was done with respect to the value obtained for unmodified U in the U context.

Table S1: List of short oligonucleotides used in this study

**RNA Sequence[a] (5′→3′)**	**Source**
<u>UUUUU</u> **U** <u>UUUUU</u>	IDT (Standard desalting)
<u>UUUUU</u> **m5U** <u>UUUUU</u>	Stanford-PAN facility (HPLC purified)
<u>UUUUU</u> **Ψ** <u>UUUUU</u>	Stanford-PAN facility (HPLC purified)
<u>UUUUU</u> **m1Ψ** <u>UUUUU</u>	Stanford-PAN facility (HPLC purified)
<u>UUUUU</u> **cU** <u>UUUUU</u>	Alnylam (HPLC purified)
<u>AAAAA</u> **U** <u>AAAAA</u>	IDT (Standard desalting)
<u>AAAAA</u> **m5U** <u>AAAAA</u>	Stanford-PAN facility (HPLC purified)
<u>AAAAA</u> **Ψ** <u>AAAAA</u>	Stanford-PAN facility (HPLC purified)
<u>AAAAA</u> **m1Ψ** <u>AAAAA</u>	Stanford-PAN facility (HPLC purified)
<u>AAAAA</u> **cU** <u>AAAAA</u>	Alnylam (HPLC purified)

[a]RNAs of sequences shown were used containing 2′-O-methyluridine (<u>U</u>), uridine (**U**), 5-methyluridine (**m5U**), pseudouridine (**Ψ**), N1-methylpseudouridine (**m1Ψ**), carbocyclic uridine (**cU**), 2′-O-methyladenosine (<u>A</u>).

Table S2. List of reagents and materials

Reagents	Source	Identifier
**Reagents, Buffers, and Enzymes**		
5 M NaCl	Invitrogen™	#AM9760G
1 M $MgCl_2$	Invitrogen™	#AM9530G
CHES, pH=10.0 ± 0.15	Boston Bioproduct, Inc.	#BB-87
HEPES, pH=7.3	Gibco™	#15630080
1xPBS, pH=7.4	Gibco™	#10010-023
10xTBE buffer	Invitrogen™	#15581044
Urea	Fisher bioreagents	#BP169-500
40wt% Acrylamide-bisacrylamide	Sigma-Aldrich	#A9926-100ML
UltraPure DTT	Invitrogen™	#15508013
N, N, N′, N′-Tetramethyl ethylenediamine	Sigma-Aldrich	#110732
Ammonium Persulfate	Thermo Scientific™	#17874
SYBR Gold Nucleic acid gel stain (10,000x)	Thermo Scientific™	#S11949
RNase A	Thermo Scientific™	#EN0531
RNase-I	Thermo Scientific™	#EN0601
RNase 4	New England Biolabs'	#M1284S
RNaseOUT™ Recombinant Ribonuclease Inhibitor	Invitrogen™	#10777019
UltraPure DNase/RNase-Free Distilled water	Invitrogen™	#10977015
96% EtOH	Thermo Scientific™	#T032021000CS
RNA Gel loading Dye (2x)	Thermo Scientific™	# R0641
3 M sodium acetate, pH=5.5	Invitrogen™	# AM9740
Glycogen, RNA grade	Thermo Scientific™	#R0551
**Commercial kits**		
Amicon Ultra-0.5 3K-Centrifugal Filter Unit	Millipore-Sigma	# UFC500324
Corning Costar Spin-X centrifuge filters, 0.22 μm	Millipore-Sigma	
RNA clean-up and concentrator-5 column	Zymo Research	# R1016
**Chemicals and Solvents**		
1,1'-Carbonyldiimidazole (CDI)	TCI	#C0119
Nicotinic acid	AK Scientific	#J52008-25g
2-Methylnicotinic acid	TCI	#M2089
DMSO	Fisher bioreagents	#BP231-100
D6-DMSO	CIL, Inc.	#DLM-10-10
D2O	CIL, Inc.	#DLM-4-100
Methanool-D4	CIL, Inc.	#DLM-24-10
Methanol	Fisher Chemical	#A412-500
2,2,2-Trifluoroethanol	Thermo Scientific™	#139751000
2,2,2-Trichloroethanol	Sigma-Aldrich	#T54801-100G
2,2-Dichloroethanol	Chem Cruz®	#SC-230697
2-Chloroethanol	Sigma-Aldrich	#185744-50G
1,3-Dichloro-2-ethanol	TCI	#D0402

Diacetin	Sigma-Aldrich	#W500615
Deuterium Chloride 35wt% solution	Sigma-Aldrich	#543047-10G
Sodium deuteroxide 40wt% solution	Sigma-Aldrich	#372072-10G
Sodium phosphaste monobasic	Sigma-Aldrich	#S5011-100G
Sodium phosphaste dibasic	Sigma-Aldrich	#S9763-100G
**Software**		
Typhoon Scanner	GE healthcare	NA
ChemDraw	Revvitycloud	NA
ImageJ	NIH	NA
MestReNova	Mestrelab	NA

Table S3. Rate constants for enzymatic cleavage[a] at the central nucleotide of RNA oligonucleotides with RNase A.

A oligonucleotide	$k_{clv(app)}$ (min^{-1})	k_2 ($\mu M^{-1} min^{-1}$)
U	28.0 (1.01) x10^{-2}	384.0 ± 13.8
m5U	1.2 (0.07) x10^{-2}	16.4 ± 0.959
Ψ	0.53 (0.05) x10^{-2}	7.26 ± 0.685
m1Ψ	0.26 (0.01) x10^{-2}	3.56 ± 0.0822

[a]Rate constants derived from 20% PAGE analysis. Conditions: 50 μM RNA, 0.00073 μM RNase A, Tris-HCl buffer (50 mM, pH 7.4) at 37 °C. Data are averages of 3 replicates; error as standard deviations are given in parentheses. See full sequences in Table 1.

References

(S1) Li, Y.; Breaker, R. R. Kinetics of RNA Degradation by Specific Base Catalysis of Transesterification Involving the 2γ-Hydroxyl Group. *J. Am. Chem. Soc.* **1999**, *121* (23), 5364–5372.

(S2) Fang, L.; Xiao, L.; Jun, Y. W.; Onishi, Y.; Kool, E. T. Reversible 2′-OH Acylation Enhances RNA Stability. *Nat. Chem.* **2023**, *15* (9), 1296–1305.

(S3) Jash, B.; Kool, E. T. Conjugation of RNA via 2′-OH Acylation: Mechanisms Determining Nucleotide Reactivity. *Chem. Commun.* **2022**, *58* (22), 3693–3696.

(S4) Ballinger, P.; Long, F. A. Acid Ionization Constants of Alcohols. II. Acidities of Some Substituted Methanols and Related Compounds. *J. Am. Chem. Soc.* **1960**, *82* (4), 795–798.
