# Supplementary material for: C‑Nucleosides Stabilize RNA by Reducing Nucleophilicity at 2′-OH
Source: ACS Cent Sci. 2025 Oct 28;11(12):2400–9. doi: 10.1021/acscentsci.5c01345 (PMC12746151; doi:10.1021/acscentsci.5c01345)
Supplement: Supplementary file 2 [file oc5c01345_si_002.pdf]

oc-2025-013457.R1

Name: Peer Review Information for "C-Nucleosides Stabilize RNA by Reducing Nucleophilicity at 2'-OH"

First Round of Reviewer Comments

Reviewer: 1

Comments to the Author

This systematic study by Kool and coworkers provides evidence that carbon substitution in ribonucleotides, particularly at the C1' position of ribose, significantly reduces the nucleophilicity of the 2'-OH group in RNA. This reduction leads to slower rates of both external electrophilic attacks (e.g., by acylating agents) and internal phosphodiester bond cleavage. The findings are particular relevant for understanding the protective effects of pseudoU and m1pseudoU modifications in both native and therapeutic RNAs. By offering quantitative data and insights into the physicochemical mechanisms underlying these effects, the study advances our understading of RNA stability and its potential for therapeutic applications.

The authors also demonstrate that pseudoU and m1-pseudoU stabilizes RNA by reducing the acidity of the 2'-OH group compared to canonical uridine, likely due to diminished inductive stabilization of the anionic form. This stabilization is consistent with broader trends observed in alcohols with reduced electronegative atoms, further supporting the hypothesis. Additionally, the study highlights the protective effects of methyl substitutions at C-5 of uridine and N1 of pseudouridine, which enhance base stacking and contribute to phosphodiester bond stability.

The broader implications of these results extend to other methylated bases, such as m5C and m6A, which are known to enhance base stacking and influence RNA reactivity. The study suggests that these modifications may similar alter 2'-OH reactivity and phosphodiester bond stability, warranting further exploration. Overall, this work provides a robust foundation for understanding the stabilizing effects of RNA modifications and offers valuable insights for the design of therapeutic RNAs. The combination of quantitative data and mechanistic insights makes this study a significant contribution to the field.

I have following points that should be considered in a revised version:

1. The inductive effects are clearly an important factor in 2'-OH reactivity. What is missing from the discussion is an examination of the effect of reactivity in light of the (pseudo)equatorial and (pseudo)axial positioning of the 2'-OH, as well as an analysis of how the effects of modifications on sugar puckering (and consequently, the orientation of the (pseudo)equatorial and (pseudo)axial 2'-OH) impact reactivity. Only pseudouridine is addressed to some extent in this regard. In this context, temperature may affect the equilibrium of ribose puckers and play a role.

2. The authors address RNA degradation mechanisms/conditions/ etc: e.g. page 2: "... This nucleophilic attack occurs spontaneously under thermal conditions, especially at elevated pH, (10) or ..." or page 4 "In general, studies of thermal degradation of unmodified mRNAs have shown that single-stranded, flexible regions are hotspots for cleavage. (16) ... " or page 8 ".... Previous studies have shown that sequence context can affect RNA cleavage rates significantly. (27)", and so on.

What I miss is mentioning/citations of some more recent studies with much more precise mechanistic considerations on spontaneous RNA degradation; see e.g.

The effect of adenine protonation on RNA phosphodiester backbone bond cleavage elucidated by deaza-nucleobase modifications and mass spectrometry. Fuchs E, Falschlunger C, Micura R, Breuker K. Nucleic Acids Res. 2019 Aug 22;47(14):7223-7234. doi: 10.1093/nar/gkz574 or e.g. The intrinsic preference of guanosine bases for cleavage-facilitating interactions with phosphodiester moieties in RNA anions revealed by base modifications and mass spectrometry. Ploner A, Mitteregger C, Glasner H, Bereiter R, Micura R, Breuker K. Nucleic Acids Res. 2025 Jun 6;53(11):gkaf494. doi: 10.1093/nar/gkaf494

3. Page 6: "Adenine is the most strongly stacking base in RNA, while uracil is the least. (24)"

I am not so sure if this is correct. G stacking can be stronger, or at least as strong, in most sequence contexts. The authors cite Ref. 24, which considers the stacking effects of

dangling ends, but I am not convinced that these studies always reflect the correct situation in double helices. See e.g., Nature of Nucleic Acid−Base Stacking: Nonempirical ab Initio and Empirical Potential Characterization of 10 Stacked Base Dimers. Comparison of Stacked and H-Bonded Base Pairs. Jiří Šponer, Jerzy Leszczyński, Pavel Hobza J. Phys. Chem. 1996, 100, 13, 5590–5596; or Stacking Interactions of Druglike Heterocycles with Nucleobases, Audrey V. Conner, Lauren M. Kim, Patrick A. Fagan, Drew P. Harding, Steven E. Wheeler, Journal of Chemical Information and Modeling, J. Chem. Inf. Model. 2025, 65, 7, 3502–3516; and many others.

Reviewer: 2

Comments to the Author

This is a highly meaningful and impactful study that elucidates the stabilizing effects of C-nucleoside modifications (e.g., pseudouridine and N1-methyl-pseudouridine) on RNA and uncovers their underlying mechanisms. The authors demonstrate that carbon substitution reduces inductive effects from nearby electronegative atoms, therefore increasing the pKa of the 2'-OH group and decreasing its nucleophilicity. This, in turn, impedes both thermal and enzymatic strand cleavage. The findings effectively bridge fundamental RNA research with applications in cutting-edge vaccines and RNA therapeutics, providing quantitative insights into how these modifications enhance RNA longevity. For instance, the authors measure the effects of C-substitutions on thermal cleavage rates (showing 10- to 32-fold stabilization in different contexts), RNase A-mediated enzymatic cleavage (up to 52-fold suppression), and 2'-OH acylation by external electrophiles. These complementary assays form a robust and convincing support, validating the enhanced stability conferred by C-nucleotides. I recommend acceptance of this paper with the following revisions.

1. The enzymatic strand cleavage is tested only with RNase A, which, while well-studied, may limit the generalizability of the conclusion. Other ribonucleases, such as RNase I, RNase R, or RNase T1, operate via different mechanisms and target distinct sites. It would be valuable to test whether the conclusions regarding C-substitutions still hold for these enzymes, potentially broadening the mechanistic insights.

2. The exploration of 2'-OH acylation and reactivity correlations with small alcohols is excellent and thought-provoking. I am curious about the potential synergistic effects of combining C-substitutions with other modifications. For example, what if N/O is replaced

by C while also substituting the 2'-OH with 2'-F? In such cases, the absence of the 2'-OH might completely prevent cleavage at that site, but could there be trade-offs in stability, immunogenicity, or helicity compared to C-substitution alone? Adding some discussions speculating on hybrid modifications would enrich the manuscript and guide future therapeutic designs.

Reviewer: 3

Comments to the Author

This manuscript by Kool and colleagues describes a series of assays with modified RNA oligonucleotides to assess the effects of chemical changes on the rate constant for RNA strand scission by internal phosphoester transfer involving the 2' oxygen atom. The experiments appear to be generally straightforward and the results appear to be sound. These findings should be of value to those seeking to improve the chemistry and stability of RNA therapeutics.

I have a few technical issues and a few minor points that I am hoping could be addressed by the authors before publication. Some of the terminology points might sound trivial, in part because most readers will understand what the authors mean, but it would be better if the wording choices were more accurate to improve readability and clarity.

Major Points

1. The authors often use the term "thermal" cleavage in place of the more commonly used "spontaneous" or "uncatalyzed". In the field, the word "thermal" is sometimes used if the chemical process being used is at a temperature that is notably elevated from room temperature of biological temperature. Because the temperatures used in the current study are more normal for RNAs, I would recommend using the more conventional terms.

2. Page 2, line 29 (and elsewhere): The authors state that the 2'-hydroxyl group is involved in the nucleophilic attack. However, under the conditions used in the current study, the hydroxyl group nucleophile makes a negligible contribution to the measured cleavage. Rather, it is the 2'-oxyanion (the deprotonated 2'-OH group) that is the predominant nucleophile. I recommend that the authors carefully check their terminology throughout to make sure they are accurate in their descriptions.

3. Page 4, Line 23 (and elsewhere): Citations for some topics often involve more modern papers, but leave out seminal papers for the topics being discussed. For example, papers by Usher or Usher and McHale probably should be cited for the instability of unstructured nucleotides.

4. Page 4, Line 36: It is not clear why the authors are citing 2'-OH pKa values when there are reasonable measurements for pKa values from oligonucleotides (such as in reference 26 – where the pKa is measured to be ~13.6 at low K+ concentrations).

5. Page 7, Line 32 (and elsewhere): The temperatures at which the buffers have been prepared (pH measured) should be noted. Although CHES is not a very problematic buffer, its pKa does change with temperature, such that the pH of the solution used in the study might be about 0.2 units lower than reported (if the buffer was prepared at 20 degrees C and the assays were conducted at 37 degrees C). Although this difference is small, it could be meaningful if someone wishes to predict the rate constants for RNA cleavage at pH 10. The pKa of Tris (page 10) is far more variable with temperature, and should also be addressed in a similar manner.

6. My largest technical concern is the apparent measurement of the remaining intact RNA as a measure of RNA degradation (see the gels in Figs. 4B and 4C). RNA is known to nonspecifically adhere to plastic Eppendorf tubes, and the concern is that the diminishing starting material could be due to sticking to the tubes. I think the assays are probably fine as measured because I think the amount of RNA used for each assay is sufficient that losing a trace amount of RNA to the plastic surface might be negligible. But, if the data exists, then measuring all RNA in the assay might be the best option.

7. It would be helpful if the authors converted nanogram values to moles or molar concentrations.

Minor Points

8. The term "in-line" should be defined on its first use.

9. Page 8, Line 26: The term "rate constants" might be better written as "rate constant".

10. Page 9, Line 30: "Thus,the" should be "Thus, the".

11. Page 9, Line 42: Reference 30 should be superscript.

12. Often, the authors write "X-fold" (with a hyphen). The hyphen is only needed when the term is used as an adjective.

13. Reference 19 has some unusual spacing in “pKa’s”. Sadly, the title of this paper is incorrectly written by the authors of this earlier paper (the apostrophe is misused), but probably should be written incorrectly here too.

Reviewer: 4

Comments to the Author

This manuscript describes the impact of carbon substitutions on the self-cleavage rate of RNA sequences. This topic is very timely, as RNA becomes used increasingly in biomedical applications and modifications can impart stability. The work is also of high quality and rigor. That being said, while the results that are uncovered are important, they are not particularly surprising given the prior literature in the area. Thus, a more suitable journal would be JACS or ACS Chemical Biology.

A few minor suggestions for the authors to consider are:

-The cleavage conditions used require a high concentration of Mg, which could impact the mechanism of cleavage. It would be helpful to discuss this potential impact

-The data for impact on RNase cleavage are certainly striking. It would be helpful to know whether this is a special attribute of the carbon-substituted nucleotides or if a similar rate dampening is experienced with other modified nucleotides.

-The correlation in Figure 5B is not particularly strong. It could be helpful to include other factors that would/wouldn't be expected to impact cleavage rate, such as pucker. This would provide context for the significance of any correlations seen.

Author's Response to Peer Review Comments:

STANFORD UNIVERSITY, Stanford, California 94305

Eric T. Kool
kool@stanford.edu

George and Hilda Daubert Professor Tel 650/724-4741

Senior Editor
ACS Central Science September 14, 2025

Dear Editor,

Submitted with this letter is our revised manuscript, entitled "C-Nucleosides Stabilize RNA by Reducing Nucleophilicity at 2′-OH", which is under consideration for publication as an Article in *ACS Central Science*. We are pleased that the reviewers as a whole were favorably disposed toward our work, and we appreciate their detailed and thoughtful comments and suggestions. In response, we have performed new experiments and have revised the text, and we believe the manuscript is improved as a result.

Below are our detailed responses to the editor's and reviewers' comments:

Reviewer: 1
Recommendation: Publish in ACS Central Science after minor revisions noted.

Reviewer's comments:
Overall, this work provides a robust foundation for understanding the stabilizing effects of RNA modifications and offers valuable insights for the design of therapeutic RNAs. The combination of quantitative data and mechanistic insights makes this study a significant contribution to the field.

Response: We thank the Reviewer.

Comment 1. The inductive effects are clearly an important factor in 2'-OH reactivity. What is missing from the discussion is an examination of the effect of reactivity in light of the (pseudo)equatorial and (pseudo)axial positioning of the 2'-OH, as well as an analysis of how the effects of modifications on sugar puckering (and consequently, the orientation of the (pseudo)equatorial and (pseudo)axial 2'-OH) impact reactivity. Only pseudouridine is addressed to some extent in this regard. In this context, temperature may affect the equilibrium of ribose puckers and play a role.

Response: This is an interesting point, and we agree that the reactivity of 2′-OH may well depend on its spatial orientation, specifically its (pseudo)equatorial versus (pseudo)axial conformation. We have now added details about the C-nucleoside vs. N-nucleoside conformations in the text (page 9).

Significantly, there is little difference in the sugar conformations of the C-nucleosides studied here relative to N-nucleoside U (Chang, Y. C.; Herath, J.; Wang, T. H. H.; Chow, C. S. Synthesis and Solution Conformation Studies of 3-Substituted Uridine and Pseudouridine Derivatives. *Bioorganic Med. Chem.* **2008**, *16* (5), 2676–2686). The published data for U, Ψ, and m1Ψ show C3′-*endo* and C2′-*endo* with very similar populations of 53:47, 48:52, and 46:54, respectively, which strongly suggests that the low reactivity of the C-nucleosides is not likely to be the result of conformational differences. As for temperature, our studies were all carried out at 37°C because our interest is in the biological relevance of the effect of the modifications on the stability of RNA.

Comment 2. The authors address RNA degradation mechanisms/conditions/etc: e.g. page 2: "… This nucleophilic attack occurs spontaneously under thermal conditions, especially at elevated pH, (10) or …" or page 4 "In general, studies of thermal degradation of unmodified mRNAs have shown that single-stranded, flexible regions are hotspots for cleavage. (16) … " or page 8 "…. Previous studies have shown that sequence context can affect RNA cleavage rates significantly. (27)", and so on.

What I miss is mentioning/citations of some more recent studies with much more precise mechanistic considerations on spontaneous RNA degradation; see e.g.

The effect of adenine protonation on RNA phosphodiester backbone bond cleavage elucidated by deaza-nucleobase modifications and mass spectrometry. Fuchs E, Falschlunger C, Micura R, Breuker K. Nucleic Acids Res. 2019 Aug 22;47(14):7223-7234. doi: 10.1093/nar/gkz574 or e.g. The intrinsic preference of guanosine bases for cleavage-facilitating interactions with phosphodiester moieties in RNA anions revealed by base modifications and mass spectrometry. Ploner A, Mitteregger C, Glasner H, Bereiter R, Micura R, Breuker K. Nucleic Acids Res. 2025 Jun 6;53(11):gkaf494. doi: 10.1093/nar/gkaf494

Response: We thank the reviewer for the suggestion. We have now added these recent references on mechanisms of spontaneous cleavage of the phosphodiester bond in RNA.

Comment 3. Page 6: "Adenine is the most strongly stacking base in RNA, while uracil is the least. (24)"

I am not so sure if this is correct. G stacking can be stronger, or at least as strong, in most sequence contexts. The authors cite Ref. 24, which considers the stacking effects of dangling ends, but I am not convinced that these studies always reflect the correct situation in double helices. See e.g., Nature of Nucleic Acid−Base Stacking: Nonempirical ab Initio and Empirical Potential Characterization of 10 Stacked Base Dimers. Comparison of Stacked and H-Bonded Base Pairs. Jiří Šponer, Jerzy Leszczyński, Pavel Hobza J. Phys. Chem. 1996, 100, 13, 5590–5596; or Stacking Interactions of Druglike Heterocycles with Nucleobases, Audrey V. Conner, Lauren M.

Kim, Patrick A. Fagan, Drew P. Harding, Steven E. Wheeler, Journal of Chemical Information and Modeling, J. Chem. Inf. Model. 2025, 65, 7, 3502–3516; and many others.

Response: We thank the reviewer for raising this important point, and we agree that stacking free energies do depend signficantly on the context. Therefore, we have revised to comment from "adenine" to "purines". We revised the manuscript on page 7 to address the reviewer's comment.

Reviewer: 2
Recommendation: Publish in ACS Central Science after minor revisions noted.
Reviewer comments:
This is a highly meaningful and impactful study that elucidates the stabilizing effects of Cnucleoside modifications (e.g., pseudouridine and N1-methyl-pseudouridine) on RNA and uncovers their underlying mechanisms. (…) These complementary assays form a robust and convincing support, validating the enhanced stability conferred by C-nucleotides.

Response: We sincerely thank the reviewer for their appreciation and for the thoughtful suggestion, which has helped strengthen our study.

Comment 1. The enzymatic strand cleavage is tested only with RNase A, which, while wellstudied, may limit the generalizability of the conclusion. Other ribonucleases, such as RNase I, RNase R, or RNase T1, operate via different mechanisms and target distinct sites. It would be valuable to test whether the conclusions regarding C-substitutions still hold for these enzymes, potentially broadening the mechanistic insights.

Response: We thank the reviewer for suggesting experiments to evaluate the generality of the Cnucleoside effect in enzymatic cleavage reactions. In response, we have expanded our study to include additional ribonucleases, RNase 4 and RNase 1, alongside RNase A. As presented in the revised manuscript (page 12) and supporting information (Materials and Methods and Figure S5), our results consistently show that the C-nucleoside pseudouridine (Ψ) reduces the rate constant of enzymatic cleavage compared to the canonical uridine (U) (Figure R1). Interestingly, the magnitude of this reduction varies depending on the ribonuclease and its catalytic mechanism.

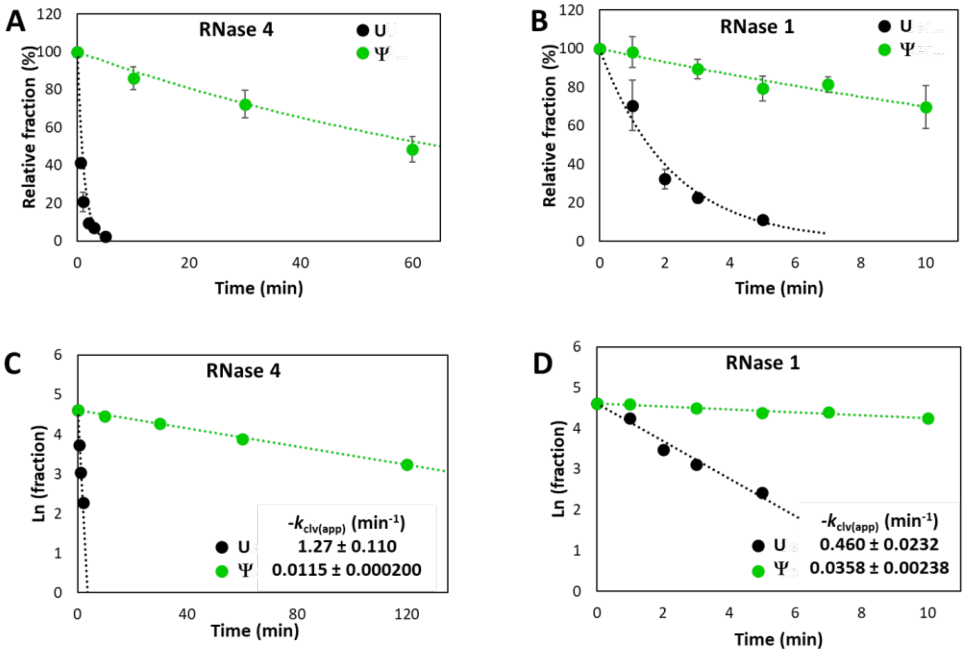

Figure R1. (A) (B) Plots showing time-dependent decay of phosphodiester bond of 50 μM 11nt RNAs by enzymatic cleavage at 37 [illegible]C with RNase 4 (1.0 U uL^{-1}) and RNase 1 (0.01 U uL^{-1}), respectively, for oligonucleotides containing U and [illegible]. (C,D) Linear plots of ln(remaining RNA fraction) vs time for unmodified and modified uridine -containing RNAs. Pseudo -first-order rate constants (k [illegible]) ar e derived from the negative slopes. Data were obtained from 3replicates with error bars showing standard deviations.

<u>Comment 2.</u> The exploration of 2'-OH acylation and reactivity correlations with small alcohols is excellent and thought-provoking. I am curious about the potential synergistic effects of combining C-substitutions with other modifications. For example, what if N/O is replaced by C while also substituting the 2'-OH with 2'-F? In such cases, the absence of the 2'-OH might completely prevent cleavage at that site, but could there be trade-offs in stability, immunogenicity, or helicity compared to C-substitution alone? Adding some discussions speculating on hybrid modifications would enrich the manuscript and guide future therapeutic designs.

<u>Response</u>: We thank the reviewer for the question. As noted, chemical modifications at the 2'-OH position and nucleobases serve distinct functional purposes in RNA therapeutics.

In response to the reviewer's suggestion, we have revised the manuscript to explicitly highlight the individual roles of C-substituted nucleobase modifications such as Ψ, and incorporated a discussion (page 19) on hybrid modification strategies to guide the rational design of nextgeneration RNA therapeutics.

Reviewer: 3

Recommendation: Major revisions required.

Reviewer comments:

This manuscript by Kool and colleagues describes a series of assays with modified RNA oligonucleotides to assess the effects of chemical changes on the rate constant for RNA strand scission by internal phosphoester transfer involving the 2' oxygen atom. The experiments appear to be generally straightforward and the results appear to be sound. These findings should be of value to those seeking to improve the chemistry and stability of RNA therapeutics.

I have a few technical issues and a few minor points that I am hoping could be addressed by the authors before publication.

Major Points

Comment 1. The authors often use the term "thermal" cleavage in place of the more commonly used "spontaneous" or "uncatalyzed". In the field, the word "thermal" is sometimes used if the chemical process being used is at a temperature that is notably elevated from room temperature of biological temperature. Because the temperatures used in the current study are more normal for RNAs, I would recommend using the more conventional terms.

Response: We thank reviewer, and we do want to avoid confusion among readers. We have revised the manuscript to relace "thermal" with "spontaneous" or "nonenzymatic" on pages 1, 2, 5, 6, 7, 8, 9, 12, 14, 17, 18, and 19.

Comment 2. Page 2, line 29 (and elsewhere): The authors state that the 2'-hydroxyl group is involved in the nucleophilic attack. However, under the conditions used in the current study, the hydroxyl group nucleophile makes a negligible contribution to the measured cleavage. Rather, it is the 2'-oxyanion (the deprotonated 2'-OH group) that is the predominant nucleophile. I recommend that the authors carefully check their terminology throughout to make sure they are accurate in their descriptions.

Response: We thank reviewer for recommending the clarification. We revised our manuscript (page 2, line 16) by describing the base catalyzed cleavage mechanism as shown in Figure 2B, where 2′-oxyanion is the predominant nucleophile that attacks the intramolecular phosphodiester bond. Note also that Figure 1C shows the oxyanion explicitly.

Comment 3. Page 4, Line 23 (and elsewhere): Citations for some topics often involve more modern papers, but leave out seminal papers for the topics being discussed. For example, papers

by Usher or Usher and McHale probably should be cited for the instability of unstructured nucleotides.

Response: We thank reviewer for the suggestion. We have now included the citation of Usher and McHale (Usher, D. A.; Mchale A. H. Hydrolytic stability of helical RNA: A selective advantage for the natural 3',5'- bond. *Proc. Nat. Acad. Sci. USA* **1976**, 73 (4), 1149-1153) for instability of unstructured nucleotides.

Comment 4. Page 4, Line 36: It is not clear why the authors are citing 2'-OH pKa values when there are reasonable measurements for pKa values from oligonucleotides (such as in reference 26 – where the pKa is measured to be ~13.6 at low K+ concentrations).

Response: We appreciate the reviewer for raising the question. We have now cited the Breaker data as well. Li and Breaker's data measured the functional pK_a cleavage at a ribonucleotide within 22nt DNA oligonucleotides containing one ribonucleotide. The determined pK_a is reasonably close to the reported pK_a of ribonucleoside 3'-monophosphates by J. Chattopadhyaya and co-workers.

Comment 5. Page 7, Line 32 (and elsewhere): The temperatures at which the buffers have been prepared (pH measured) should be noted. Although CHES is not a very problematic buffer, its pKa does change with temperature, such that the pH of the solution used in the study might be about 0.2 units lower than reported (if the buffer was prepared at 20 degrees C and the assays were conducted at 37 degrees C). Although this difference is small, it could be meaningful if someone wishes to predict the rate constants for RNA cleavage at pH 10. The pKa of Tris (page 10) is far more variable with temperature, and should also be addressed in a similar manner.

Response: We are thankful to reviewer for raising this vital point. In this study, we utilized a commercially available CHES buffer with a reported pH of 10.0 ± 0.15. Prior to initiating the experiments, the pH of the buffer solution was independently verified to be 10.19 at 37 °C. This has now been made clear in the experimental description.

Comment 6. My largest technical concern is the apparent measurement of the remaining intact RNA as a measure of RNA degradation (see the gels in Figs. 4B and 4C). RNA is known to nonspecifically adhere to plastic Eppendorf tubes, and the concern is that the diminishing starting material could be due to sticking to the tubes. I think the assays are probably fine as measured because I think the amount of RNA used for each assay is sufficient that losing a trace amount of

RNA to the plastic surface might be negligible. But, if the data exists, then measuring all RNA in the assay might be the best option.

Response: We appreciate the reviewer's concern regarding potential sample loss due to RNA adherence, and we have revised the supporting methods and materials section addressing the point. In the kinetic studies of RNA cleavage, oligonucleotide concentrations exceeding 50 µM were employed, which substantially reduces the surface-to-RNA ratio and thereby minimizes nonspecific adsorption to Eppendorf tube walls. Additionally, the use of 50 mM CHES buffer (pH 10.2) provides electrostatic shielding, further mitigating RNA-surface interactions. To ensure accurate quantification of the remaining RNA fraction at each time point, reaction mixtures were gently vortexed and centrifuged prior to quenching, thereby promoting homogeneity and minimizing sampling error. Finally, all controls and comparisons were treated equally, so relative rate effects should be unaffected.

Comment 7. It would be helpful if the authors converted nanogram values to moles or molar concentrations.

Response: We appreciate reviewer for the suggestion. We have revised the manuscript with molar concentrations where only nanogram quantities were mentioned.

Minor Points

Comment 8. The term "in-line" should be defined on its first use.

Comment 9. Page 8, Line 26: The term "rate constants" might be better written as "rate constant".

Comment 10. Page 9, Line 30: "Thus,the" should be "Thus, the".

Comment 11. Page 9, Line 42: Reference 30 should be superscript.

Comment 12. Often, the authors write "X-fold" (with a hyphen). The hyphen is only needed when the term is used as an adjective.

Comment 13. Reference 19 has some unusual spacing in "p K a's". Sadly, the title of this paper is incorrectly written by the authors of this earlier paper (the apostrophe is misused), but probably should be written incorrectly here too.

Response: We have made these changes as suggested.

Reviewer: 4

Recommendation: Publish elsewhere Reviewer comments:

This manuscript describes the impact of carbon substitutions on the self-cleavage rate of RNA sequences. This topic is very timely, as RNA becomes used increasingly in biomedical applications and modifications can impart stability. The work is also of high quality and rigor. That being said, while the results that are uncovered are important, they are not particularly surprising given the prior literature in the area. Thus, a more suitable journal would be JACS or ACS Chemical Biology.

Response: While we respectifully disagree with the suggestion of different journals, we are thankful to the reviewer for the appreciation and helpful suggestions. We have revised the manuscript to address the comments; the changes are highlighted by yellow in the revised manuscript.

A few minor suggestions for the authors to consider are:

-The cleavage conditions used require a high concentration of Mg, which could impact the mechanism of cleavage. It would be helpful to discuss this potential impact

Response: We appreciate reviewer for highlighting the potential influence of Mg^{2+} concentration on the cleavage mechanism. In our study, a Mg^{2+} concentration of 10 mM was essential to achieve efficient RNA cleavage, primarily by increasing the ionic strength under the chosen reaction conditions. While this concentration was optimized to support robust cleavage activity, we acknowledge that elevated Mg^{2+} levels may influence both the rate and mechanistic pathway of cleavage. This potential impact has been considered and addressed in the revised manuscript (page 7).

Magnesium ions are known to play a dual role in RNA cleavage; they catalyze phosphodiester bond cleavage by stabilizing the transition state and activating the 2′-OH nucleophiles, while simultaneously promoting RNA folding into compact, protective structures. At elevated concentrations from 0.005 to 0.05 M, Mg^{2+} can accelerate cleavage by enhancing nucleophilic attack and reducing activation energy (Li, Y.; Breaker, R. R. Kinetics of RNA Degradation by Specific Base Catalysis of Transesterification Involving the 2γ-Hydroxyl Group. *J. Am. Chem. Soc.* **1999**, *121* (23), 5364–5372.). However, it may also induce conformational changes that shield cleavage sites or alter enzyme accessibility. Recent studies have described a “Goldilocks” behavior, wherein RNA stability peaks at intermediate Mg^{2+} levels, too little Mg^{2+} leads to unfolded, labile RNA, while excessive Mg^{2+} (> 1 M) can override protective folding and increase degradation rates (Guth-Metzler, R.; Mohamed, A. M.; Cowan, E. T.; Henning, A.; Ito, C.;

Frenkel-Pinter, M.; Wartell, R. M.; Glass, J. B.; Williams, L. D. Goldilocks and RNA: where Mg^{2+} concentration is just right. *Nucleic Acids Res.* **2023**, *51* (8), 3529–3539).

-The data for impact on RNase cleavage are certainly striking. It would be helpful to know whether this is a special attribute of the carbon-substituted nucleotides or if a similar rate dampening is experienced with other modified nucleotides.

<u>Response</u>: We thank the reviewer for the question. Our work focused on uracil base modifications, which are among the most prevalent in the transcriptome. In addition to C-nucleoside structure, we also examined the effects of C-5 methylation, studying three known modified bases in two contexts as well as one modified ribose. At the end of the manuscript, we do speculate on how the current data may relate to other modifications such as m5C or m6A, but study of those would require a different experimental setup (due to cleavage occurring on either side of the modification) and was beyond the scope of the current study.

Ribose modifications can, of course, strongly inhibit or prevent RNA cleavage; for instance, 2′-O-methyl and 2′-fluoro substitutions fully inhibit RNase cleavage by sterically and electronically protecting the 2′-OH group (Tong, A.; Leylek, R.; Herzner, A.; Rigas, D.; Wichner, S.; Blanchette, C.; Tahtinen, S.; Kemball, C. C.; Mellman, I.; Haley, B.; Freund, E. C.; Delamarre, L. Nucleotide modifications enable rational design of TLR7-selective ligands by blocking RNase cleavage. *J. Exp. Med.* **2024**, *221* (2), e20230341).

In contrast to this, our work primarily addresses base modifications, which are more remote from the reactive 2′-OH group. Nucleobase modifications have the potential to attenuate cleavage through stacking interactions, steric hindrance, and inductive effects (Harcourt, E. M.; Kietrys, A. M.; Kool, E. T. Chemical and structural effects of base modifications in messenger RNA. *Nature* **2017**, *541*, 339–346).

-The correlation in Figure 5B is not particularly strong. It could be helpful to include other factors that would/wouldn't be expected to impact cleavage rate, such as pucker. This would provide context for the significance of any correlations seen.

<u>Response</u>: We appreciate this suggestion, and have added a figure to the Supporting data file. Although the sugar pucker of U/□□ /m1□ are very nearly the same (see revised page 9), we have re-examined circular dichroism (CD) spectra for the 10 oligonucleotides used in the study. The CD spectral parameters associated with ribose conformational states, particularly ellipticity near 260–280 nm, were analyzed to determine whether nucleotide substitutions influence global sugar pucker and thereby modulate RNA backbone geometry. Below is the modified Figure, overlaying the ellipticity data for all oligomers in comparison with unmodified uridine in the U context. The

plot clearly illustrates that no marked conformation changes were noted in comparison to the large differences observed in rate constants of acylation and strand cleavage.

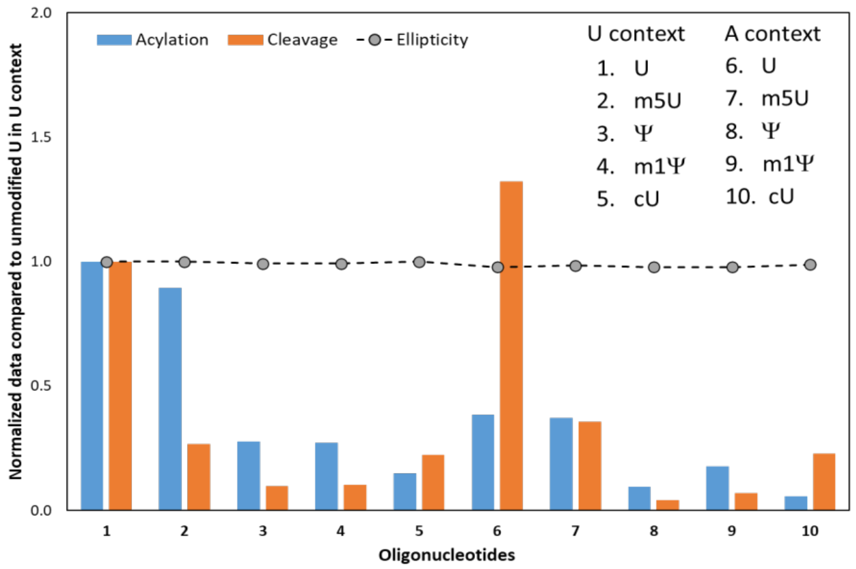

Figure R2. Comparison of normalized rate constants for the acylation and spontaneous cleavage of modified nucleotides in the RNA oligonucleotides with corresponding ellipticity shift. Normalization was done with respect to the value obtained for unmodified U in the U context.

Editor's formatting comments:

Supporting Information: If the manuscript is accompanied by any Supporting Information for Publication, a brief description of the supplementary material is required in the manuscript, before the reference list.

A brief description of Supporting Information is now included on page 20.

Synopsis: ACS Central Science requires a brief synopsis.

We have now included a Synopsis on page 1.

TOC Graphic: Please label as "TOC Graphic" in the manuscript.

The TOC Graphic is now labelled appropriately.

With these changes, we hope you will find our manuscript acceptable for publication.

Sincerely,

Eric T. Kool
Department of Chemistry and Stanford Cancer Institute

oc-2025-013457.R2

Name: Peer Review Information for "C-Nucleosides Stabilize RNA by Reducing Nucleophilicity at 2'-OH"

Second Round of Reviewer Comments

Reviewer: 2

Comments to the Author

My previous concerns are well addressed in the revised version and I recommend publish this paper as the current form.

Reviewer: 1

Comments to the Author

All my concerns have been addressed in a satisfactory manner by the authors.

Author's Response to Peer Review Comments:

STANFORD UNIVERSITY

Stanford, California 94305

Eric T. Kool
George and Hilda Daubert Professor

Senior Editor

ACS Central Science

October 18, 2025

DearEditor:

Submitted with this letter is our re-revised manuscript, entitled “C-Nucleosides Stabilize RNA by Reducing Nucleophilicity at 2′-OH”, which is provisionally accepted for publication as an Article in *ACS Central Science*.

We corrected the format of page numbering in the Supporting file as requested by your office.

During re-checking of the data, we discovered an arithmetic error in the rate constants for acylation (Tables 2 and 3), resulting in the decimal points being shifted for those cases. This makes no difference to the analysis or conclusions in the work, which compared relative (not absolute) rates. We have replaced these Tables with corrected versions, along with changing the y-axis scale on Figs. 5E and S9 to reflect this numeric change.

Gratifyingly, the numeric change also made the data comparing RNA and small alcohols more internally consistent. A paragraph in the Discussion section previously addressed this, and so we edited it slightly for accuracy.

We have uploaded both a marked version with edits in yellow, along with a clean version.

With these changes, we hope you will find our manuscript acceptable for publication.

Sincerely yours,

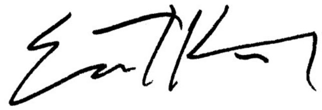

Eric T. Kool

Department of Chemistry and Stanford Cancer Institute
Stanford University
